# What are the perspectives for blood donations and blood component transfusion worldwide? A systematic review of time series studies

**DOI:** 10.1590/1516-3180.2019.0415.R1.06112019

**Published:** 2020-04-22

**Authors:** Elias Melo de Oliveira, Ilka Afonso Reis

**Affiliations:** I MSc. Healthcare Services Manager, Department of Preventive and Social Medicine, Universidade Federal de Minas Gerais (UFMG), Belo Horizonte (MG), Brazil.; II PhD. Professor, Department of Statistics, Universidade Federal de Minas Gerais (UFMG), Belo Horizonte (MG), Brazil.

**Keywords:** Blood transfusion, Aging, Blood supply [subheading], Blood donation, Systematic review, Blood demand, Time series

## Abstract

**BACKGROUND:**

Analysis of the literature suggests that changes relating to blood donations and blood component transfusion are occurring due to the aging of the population.

**OBJECTIVE:**

To gain better understanding of the demand and supply of these inputs over time, and to identify the main associated demographic characteristics.

**DESIGN AND SETTING:**

Systematic review conducted on time series relating to blood donations and blood component transfusions worldwide.

**METHODS:**

A systematic review of the literature was conducted based on articles that presented time series relating to blood donation or blood component transfusion.

**RESULTS:**

We found 1,814 articles. After the deletion process, only thirteen were read. Overall, these suggested that there is increasing demand for blood components and decreasing donation. The existence of seasonality regarding blood donation was pointed out. Men usually donated more blood and demanded more blood components than women. Approximately 50% of blood transfusions were performed in people aged ≥ 60 years.

**CONCLUSIONS:**

This analysis on articles that presented time series relating to blood donations and blood component transfusion showed that aging of the population was the main factor associated with the increasing demand for blood and the decreasing supply of blood.

## INTRODUCTION

Because of the growing aging of the population, the demand for healthcare services, especially hospital services, is expected to increase.^1^ At the same time, growing demand for blood components will accompany this increase.^2^^,^^3^

In addition to the costs and risks associated with the use of blood components, there has been much discussion in the literature regarding changes to the demand and supply of blood components, consequent to the aging of the population.^3^^,^^4^^,^^5^

Faced with the new projected demographic context, there are two healthcare problems: reduced blood donations and increased use of blood components, because the elderly population is the one that proportionally uses more of this input.^1^^,^^2^^,^^3^^,^^5^^,^^6^^,^^7^

Understanding the context and perspectives of blood supply and demand for blood components is essential, to make it possible to define strategies relating to these inputs.

## OBJECTIVE

The objective of this study was to conduct a systematic review of the literature, with a search for studies on time series relating to blood donation rates and blood component transfusions, along with the demographic characteristics of the blood donor and blood recipient populations.

## METHODS

This was a systematic review study that was conducted in accordance with the Preferred Reporting Items For Systematic Methodology in Meta-Analyses (PRISMA). The review protocol was previously registered with PROSPERO under the identification code: CRD42019118995.

To identify articles, a search on this topic was carried out in January 2019 in the national and international literature, for papers published between January 1, 2005, and December 31, 2018. Articles were selected through a four-stage process.

In the first stage, three bibliographic searches were performed. The first consisted of a search using the descriptors “blood donation” and “time series” in the SciELO, PubMed and Medline databases. The second consisted of a search using the descriptors “blood transfusion” and “time series” in the SciELO, PubMed and Medline databases. The third consisted of a search in the gray literature on this subject based on the literature that is considered classic for the subject and its references.

The second stage of article selection involved exclusion of texts that were found in duplicate.

The third stage of article selection involved exclusion of studies that did not present the research topic, either in the title or in the abstract.

Lastly, the fourth stage of article selection involved assessment of the eligibility of the texts that met the objectives of this review, i.e. studies that presented a time series for blood donation or blood component transfusion.

### Results presentation

The results were presented in table form. To extract the data from the articles, an instrument for summarizing the main characteristics of the selected articles was elaborated. The data extracted were the following: author and year of publication; place of publication; type of outcome analyzed (transfusion or donation); and number of study participants (donors and/or patients).

Next, the main findings of the articles that were selected to form part of the time series were highlighted. These were the following: the observed trends (decrease or increase) for blood supply and blood component demand; and the observed seasonality of blood supply and blood component demand.

The demographic characteristics (gender and age) of the blood donor and blood component recipient populations presented in the selected articles were then explored. In this regard, we sought to answer two questions relating to blood supply: Who donates more blood: men or women? Young or old? Two questions relating to blood component transfusions were also answered: Who receives more blood components: men or women? Young or old?

Lastly, for each study, the factors associated with the outcomes that were found were identified, especially demographic and epidemiological factors.

### Analysis of results

The studies were analyzed separately by two researchers and any discrepancies found were addressed by a third researcher. Initially, the main characteristics of the articles were presented, such as the representativeness of the countries and the journals with the largest number of articles published.

Next, the main outcomes found in the articles regarding the time series were analyzed. These were the trends and seasonality of blood supply and use of blood components. Lastly, the main demographic and epidemiological findings that explained the behavior of the series presented in each study were also analyzed.

## RESULTS

### Selection of texts

We found 241 articles (197 in PubMed, one in SciELO and 43 Medline) in the first search; 1,559 articles (809 in PubMed, 22 in SciELO and 728 in Medline) in the second search; and fourteen articles in the third search, thus totaling 1,814 articles gathered in the first stage of article selection.

The second stage of article selection involved exclusion of duplicate articles. At this stage, 720 articles were excluded, which left 1,094 articles for assessment of eligibility.

The third stage of article selection involved exclusion of articles that did not present the research topic in the title or abstract. In this phase, 1,072 articles were excluded, thus leaving 22 articles for assessment of eligibility.

**Figure 1.**
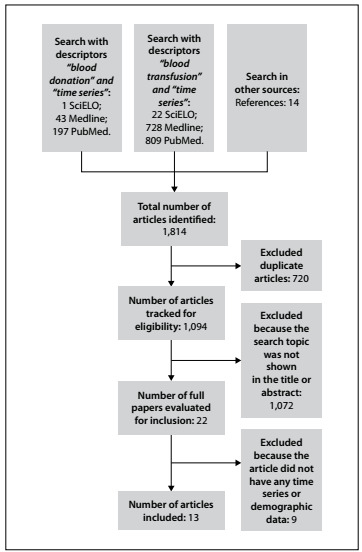
Flowchart for identifying and selecting articles for this systematic review.

In the fourth stage of article selection, the articles that met the review objectives were selected, i.e. articles that presented a time series for blood donation or blood component transfusion. At this stage, thirteen articles were selected and nine articles were excluded. The articles thus selected and the justifications for excluding articles at this stage are shown in Figure 1.

### Features of articles

Regarding the general characteristics of the articles, the oldest dated from 2004, and the most recent from 2018. The journal with the largest number of articles published was Transfusion (seven articles). Germany was the place from which the largest number of articles was selected. The number of subjects in the studies ranged from around 70,000 to around four million. The length of follow-up of the time series ranged from one to 61 years.

Eight articles presented results relating to donation and ten articles presented results relating to blood component transfusions. Eleven articles presented results relating to trend and two, seasonality of the time series.

In total, 16 countries were represented in the thirteen articles included in this review. The identification and main characteristics of the articles are shown in Table 1.^8^^,^^20^

**Table 1.** Identification and characteristics of the articles

Article code	Author	Country	Outcome	Number of individuals
1	Ali et al.^8^	Finland	Transfusion	---
2	Borkent-Raven et al.^9^	Netherlands	Transfusion	290,043
3	Borkent-Raven et al.^10^	Netherlands	Donation/Transfusion	---
4	Carneiro-Proietti.^11^	Brazil	Donation	426,142
5	Crawford et al.^12^	US	Donation	---
6	Currie et al.^13^	UK	Donation/Transfusion	70,208
7	Drackley et al.^14^	Canada	Donation/Transfusion	209,515
8	Greinacher et al.^15^	Germany	Donation/Transfusion	---
9	Greinacher et al.^16^	Germany	Donation/Transfusion	---
10	Greinacher et al.^17^	Germany	Transfusion	95,477
11	Oliveira et al.^18^	Brazil	Donation	1,246,462
12	Pfuntner et al.^19^	US	Transfusion	---
13	Volken^20^	Switzerland	Donation/Transfusion	3,931,955

### Outcomes

The most common temporal outcome regarding blood donations was reduced supply. With regard to seasonality, there were significant reductions in blood donation over holiday periods or holiday weeks. Regarding demographic variables, men generally donated more blood than women, in all the articles that presented such a comparison.^9^^,^^11^^,^^12^^,^^13^^,^^14^^,^^15^^,^^16^^,^^17^^,^^20^ People aged between 17 and 35 years were the ones who donated the most blood.

Regarding blood component transfusions, the most common temporal outcome was an increasing frequency of transfusions performed with increasing age of the population.^8^^,^^10^^,^^13^^,^^14^^,^^15^^,^^16^^,^^17^^,^^19^^,^^20^ The series analyzed in this study did not show any seasonality component. In general, men received more blood components than women.^9^^,^^11^^,^^12^^,^^13^^,^^14^^,^^15^^,^^16^^,^^17^^,^^20^ On the other hand, people aged 65 and over received the largest number of blood component transfusions.

The main outcomes relating to demographic characteristics and to blood donations and blood component transfusions are presented in Table 2.

**Table 2.** Temporal components and demographic characteristics of blood donation and blood component transfusion series

Article code	Trend	Seasonality	Gender	Age
1	Increased demand and decreased offer	---	---	Older people demanded more than the other groups
2	---	---	Men demanded more than women	Older people demanded more than the other groups (≥ 65 years demanded 42.6% of transfusions)
3	Increased demand and decreased offer	---	---	Older people demanded more than the other groups (≥ 65 years demanded 59% of transfusions)
4	---	---	Men donated more than women (65.4% of donations were made by men)	Youth group donated more than other groups (group 25-35 years made 35.5% of donations; group ≥ 55 years made 2.9% of donations)
5	Decreased offer	Among young people, peak in April and lowest between Sep and Nov; among the elderly, peaks in January and summer	Men donated more than women (57.4% of donations were made by men)	Youth group donated more than other groups (18-24 years)
6	Increased demand and decreased offer	---	Men donated more than women; women demanded more than men (56% of transfusions were performed on women)	Older people demanded more than the other groups (≥ 70 years demanded 46% of transfusions)
7	Increased demand of greater magnitude than increased supply.	---	Men donated and demanded more than women	Youth group donated more than other groups (group 17-35 years)
8	Increased demand and decreased offer	---	Men donated and demanded more than women	Youth group donated more than other groups (group 20-29 years); older people demanded more than the other groups (≥ 60 years)
9	Increased demand and decreased offer	---	Men donated more than women	Youth group donated more than other groups; older people demanded more than the other groups (≥ 65 years)
10	Increased demand and decreased offer	---	Men donated and demanded more than women (52% and 54.2% respectively)	Youth group donated more than other groups (group between 20 and 44 years made 30.9% of donations); older people demanded more than the other groups (≥ 65 years required 47% of transfusions)
11	Stationary series	Lower in weeks with holidays	---	---
12	Increased demand and decreased offer	---	---	Older people demanded more than the other groups
13	Increased demand and decreased offer	---	Men donated and demanded more than women (57.3% of transfusions)	Youth group donated more than other groups (18-24 years); older people demanded more than the other groups (≥ 65 years required 48% of transfusions)

Regarding the factors associated with the outcomes analyzed, ten articles presented possible epidemiological explanatory factors^8^^,^^9^^,^^10^^,^^12^^,^^13^^,^^14^^,^^16^^,^^17^^,^^19^^,^^20^ and eleven presented possible demographic explanatory factors.^8^^,^^9^^,^^10^^,^^11^^,^^12^^,^^13^^,^^14^^,^^15^^,^^16^^,^^17^^,^^20^ Increasing numbers of surgical procedures, greater circulatory system care and larger numbers of neoplasms were the main explanatory reasons found among the articles.

Population aging and migration were the main demographic factors associated with the outcomes. The main factors associated with the outcomes presented in the articles assessed here are organized in Table 3.

**Table 3.** Epidemiological and demographic factors associated with outcomes

Article Code	Epidemiological factors	Demographics
1	Increase in numbers of surgical procedures, procedures relating to the circulatory system and neoplasms	Ageing population
2	Increase in numbers of procedures relating to neoplasms and the circulatory system, pregnancy and childbirth	Ageing population
3	---	Ageing population
4	Number of donors women significantly lower than donors men, due to iron deficiency in population and neoplasms	Largest donations by replacement donors
5	Disasters and terrorist attacks significantly increase the number of donations	Ageing population
6	In the transfused group, 67% received an average of 2 transfusions while 33% received 5 or more transfusions	Ageing population
7	Transfusions in women are associated with pregnancy, childbirth and other events relating to the reproductive system	Ageing population
8	---	Ageing population; migration
9	Increase in number of surgical procedures relating to the circulatory system; family, pregnancy, iron deficiency and work were mainly responsible for non-donation among women	Ageing population
10	Increase in number of medical interventions	Ageing population
11	---	---
12	Increase in number of medical interventions	---
13	Increase in number of medical procedures and interventions relating to older people	Ageing population

Based on this review of the literature on time series relating to blood donations and blood component transfusions, it was possible to identify population aging as the main factor responsible for the context of the growing demand for blood component transfusions and the drop in blood donations.

## DISCUSSION

The main finding of this study was that increased demand for blood component transfusions was occurring simultaneously with reductions in blood donation rates. This temporal perspective points towards future blood shortages, due to the increasing size of the elderly population and the reduction in size of the population that is able to donate.

The presence of chronic diseases such as those relating to the circulatory system and neoplasms is inherent in elderly populations. In addition, technological development allows for longer life, but at the cost of increased numbers of surgical procedures. Both of these epidemiological contexts are associated with higher demand for blood component transfusion, as addressed here.

On the other hand, the change in the age structure of the populations studied in the articles of this review, in which the growth of the elderly population and the consequent reduction of the young population were evident, will bring with it reductions of the donor population. First, because the population eligible for donation will decrease. Second, because within the donor population there will also be significant changes. Replacement donors are among the older members of this population and spontaneous donors among the younger.^14^

Soon, the group of replacement donors will no longer be able to donate. At the same time, due to the steadily declining birth rate, the young population will not be sufficient to meet the estimated high demand.

In view of this context, several studies in the literature have addressed the ways in which new blood donors are recruited, to identify improvements in the efficiency of techniques and strategies.^21^^,^^22^ Stimuli towards increasing the replacement donor group have also been addressed.^23^

Before this, however, there is a need to focus on reducing the number of blood component transfusions. Alternative blood transfusion strategies that are safe, efficient and cost-effective have been considered in the literature. The use of these strategies is more frequent among organizations offering healthcare services. Bloodless medicine and surgery programs that seek to reduce blood use have significantly reduced the number of transfusions. These actions are implemented through protocol changes, incorporation of new technologies and more accurate control over blood component requests.^3^^,^^4^^,^^5^^,^^7^^,^^24^^,^^25^^,^^26^

Despite the large number of articles published in the databases that were searched in this study, few studies have addressed blood donation and transfusion rates from the perspective of statistical time series analysis.

In addition, the authors of the various studies presented their results differently, which makes it difficult to analyze the studies in an integrated manner. With regard to demographic variables, for example, there is a distinction between the countries from which the data were extracted. In Brazil, elderly people are defined as those aged 60 years and over. On the other hand, in Europe, elderly people are generally considered to be those aged 65 and over.

## CONCLUSION

Because of the estimated demographic context and the impossibility of changes to the age structure of populations, and because so few studies have evaluated time series relating to blood donations and blood component transfusions, further studies are essential. In particular, there is a need for studies addressing epidemiological, clinical and demographic characteristics. The present study makes a contribution towards more accurate perceptions of blood donations and blood component transfusions worldwide.
